# The 11S Proteasomal Activator REGγ Impacts Polyglutamine-Expanded Androgen Receptor Aggregation and Motor Neuron Viability through Distinct Mechanisms

**DOI:** 10.3389/fnmol.2017.00159

**Published:** 2017-05-24

**Authors:** Jill M. Yersak, Heather L. Montie, Erica S. Chevalier-Larsen, Yuhong Liu, Lan Huang, Martin Rechsteiner, Diane E. Merry

**Affiliations:** ^1^Department of Biochemistry and Molecular Biology, Thomas Jefferson UniversityPhiladelphia, PA, United States; ^2^Department of Microbiology and Immunology, Thomas Jefferson UniversityPhiladelphia, PA, United States; ^3^Department of Biochemistry, University of Utah School of MedicineSalt Lake City, UT, United States

**Keywords:** proteasome, PA28, androgen receptor, polyglutamine, neurodegeneration

## Abstract

Spinal and bulbar muscular atrophy (SBMA) is caused by expression of a polyglutamine (polyQ)-expanded androgen receptor (AR). The inefficient nuclear proteasomal degradation of the mutant AR results in the formation of nuclear inclusions containing amino-terminal fragments of the mutant AR. PA28γ (also referred to as REGγ) is a nuclear 11S-proteasomal activator with limited proteasome activation capabilities compared to its cytoplasmic 11S (PA28α, PA28β) counterparts. To clarify the role of REGγ in polyQ-expanded AR metabolism, we carried out genetic and biochemical studies in cell models of SBMA. Overexpression of REGγ in a PC12 cell model of SBMA increased polyQ-expanded AR aggregation and contributed to polyQ-expanded AR toxicity in the presence of dihydrotestosterone (DHT). These effects of REGγ were independent of its association with the proteasome and may be due, in part, to the decreased binding of polyQ-expanded AR by the E3 ubiquitin-ligase MDM2. Unlike its effects in PC12 cells, REGγ overexpression rescued transgenic SBMA motor neurons from DHT-induced toxicity in a proteasome binding-dependent manner, suggesting that the degradation of a specific 11S proteasome substrate or substrates promotes motor neuron viability. One potential substrate that we found to play a role in mutant AR toxicity is the splicing factor SC35. These studies reveal that, depending on the cellular context, two biological roles for REGγ impact cell viability in the face of polyQ-expanded AR; a proteasome binding-independent mechanism directly promotes mutant AR aggregation while a proteasome binding-dependent mechanism promotes cell viability. The balance between these functions likely determines REGγ effects on polyQ-expanded AR-expressing cells.

## Introduction

Spinal and bulbar muscular atrophy (SBMA) is an X-linked neurodegenerative disease that is caused by expansion of a polyglutamine (polyQ)-encoding CAG repeat in exon 1 of the androgen receptor (AR) gene (La Spada et al., 1991). The disease is characterized by muscle atrophy and the loss of motor neurons from the spinal cord and brainstem (Sobue et al., 1989). Moreover, as in other polyQ diseases, remaining motor neurons are marked by the presence of neuronal intranuclear inclusions (NII) of the mutant polyQ-expanded protein (Li et al., 1998). In SBMA, NII are comprised of the amino-terminal portion of expanded AR; in addition, retention of the mutant AR in the cytoplasm prevents its proteolytic cleavage (Montie et al., 2009), suggesting that the mutant AR is aberrantly cleaved within the nucleus (Li et al., 1998; Li M. et al., 2007).

Model systems of SBMA reveal the requirement for both androgen (testosterone or dihydrotestosterone—DHT) binding (Katsuno et al., 2002; Takeyama et al., 2002; Walcott and Merry, 2002; Chevalier-Larsen et al., 2004) and nuclear entry of the AR for disease (Takeyama et al., 2002; Montie et al., 2009; Nedelsky et al., 2010). Moreover, while nuclear localization of the mutant AR is necessary, it is not sufficient, as nuclear AR still requires hormone binding to cause AR aggregation and toxicity (Montie et al., 2009; Nedelsky et al., 2010). The nucleus of the cell differs from the cytoplasm in its degradation capabilities, lacking lysosomal and autophagic degradation machineries. Thus, proteins that are not exported to the cytoplasm for degradation must be degraded by nuclear proteasomes. This difference may explain the requirement for AR nuclear localization in its toxicity, as inhibition of autophagy in cells expressing cytoplasmically localized mutant AR removes the protection that this localization otherwise affords (Montie and Merry, 2009; Montie et al., 2009). The limited degradation machinery within the nucleus could expose this compartment to higher levels of cellular stress in the presence of mutant AR.

Ubiquitin-proteasome system (UPS) function is central to the molecular pathogenesis of many neurodegenerative diseases. For example, both ubiquitin and components of the UPS are found within nuclear inclusions in SBMA cell and mouse models, as well as in patient autopsy material, implicating deficits in expanded AR proteasomal degradation both *in vitro* and *in vivo* (Li et al., 1998; Stenoien et al., 1999; Abel et al., 2001; Bailey et al., 2002).

The proteasome is a dynamic protein complex that contains a 20S core, which is composed of two outer α rings and two inner catalytic β rings, each composed of seven subunits (α_7_, β_7_, β_7_, α_7_; Orlowski and Wilk, 2003). The three proteolytic active sites present in the inner β rings hydrolyze peptide bonds with chymotrypsin-like (CT-L), trypsin-like (T-L), and peptidylglutamyl-preferring hydrolytic (PGPH) or caspase-like (C-L) activities. The 20S proteasomal core is catalytically activated by the binding of a proteasomal activator to its outer α rings. The known proteasomal activators include the 19S, 11S and PA200 activators, which can bind to the 20S proteasome core in multiple combinations on either end, forming both homotypic and hybrid proteasomes (Tanahashi et al., 2000). The 11S proteasomal activator PA28 (REG) is found as three distinct isoforms, REGα, β and γ, which bind to and activate the proteasome in an ATP- and ubiquitin-independent manner (Gray et al., 1994). REGα and REGβ, which form heteroheptameric rings (α3, β4) and activate all three proteolytic activities of the 20S proteasome, are confined to the cytoplasm. In contrast, REGγ, which is largely confined to the nucleus, binds to the 20S core as a homoheptameric ring and primarily activates only its T-L activity (Realini et al., 1997). The limited proteasome-activating function of REGγ is largely conferred by lysine 188, since a mutation of this lysine to an acidic amino acid (K188E, K188D) alters its function to promote the activation of all three proteasomal catalytic activities (Li et al., 2001).

The function of REGγ as an 11S proteasomal activator plays a role in a variety of cellular processes. For example, REGγ promotes the degradation of the cell cycle regulatory proteins p21^WAF/CIP1^, p16^INK4A^, p19^ARF^ and oncogene SRC3 in an ATP- and ubiquitin-independent manner, thereby affecting cell cycle regulation and cell proliferation (Li et al., 2006; Li X. et al., 2007; Chen et al., 2007). Gene ablation of REGγ in a mouse model results in significantly reduced body size and REGγ-deficient embryonic fibroblasts fail to enter S phase of the cell cycle (Murata et al., 1999; Barton et al., 2004), further implicating REGγ in cell proliferation and cell cycle regulation. Other nuclear targets of REGγ include SIRT1 and DBC1 (Dong et al., 2013; Magni et al., 2014). In addition, while other REGγ substrates are unknown, REGγ was also shown to modulate the homeostasis of several distinct nuclear bodies, including Cajal bodies (Cioce et al., 2006) and nuclear speckles (Baldin et al., 2008). An additional role for REGγ that is independent of its binding to the 20S proteasomal core involves its direct stimulation of MDM2-dependent polyubiquitination of p53, resulting in p53 degradation by the 26S proteasome, thereby promoting cell survival (Zhang and Zhang, 2008).

Here we present data demonstrating that REGγ influences polyQ-expanded AR aggregation and toxicity in SBMA cell and motor neuron models through distinct proteasome binding-dependent and -independent mechanisms. We discovered that REGγ overexpression increases ARQ112 aggregation in PC12 cells in a proteasome activating- and binding-independent manner. REGγ interacts with expanded AR, decreasing its polyubiquitination, possibly by decreasing its interaction with the E3 ligase MDM2. REGγ overexpression also failed to protect PC12 cells from DHT-dependent AR toxicity. On the other hand, REGγ overexpression rescued ARQ112-expressing motor neurons from DHT-dependent cell death in a proteasome binding-dependent manner. Evaluation of several known and candidate REGγ substrates revealed that one candidate REGγ substrate, RNA splicing factor SC35 (Srsf2; Fu and Maniatis, 1992; Shepard and Hertel, 2009), is elevated upon polyQ-expanded AR expression, further elevated upon DHT treatment, and is reduced by REGγ overexpression. Moreover, reducing SC35 levels proved to be protective in both cell models of SBMA. Altogether, our results suggest that REGγ may play a neuroprotective role in cell types in which its proteasome-activating functions supersede its proteasome binding-independent functions.

## Materials and Methods

### Cell Culture and Reagents

Tet-On PC12 cells expressing either ARQ112 or ARQ10, were previously created in our laboratory (Walcott and Merry, 2002). Cells were maintained in normal growth media (Dulbecco’s modified Eagle’s medium with 10% heat-inactivated horse serum, 5% heat-inactivated fetal bovine serum, 2 mM L-glutamine, 100 units/ml penicillin/streptomycin, 200 μg/ml hygromycin (Invitrogen, Carlsbad, CA, USA), 100 μg/ml G418 (Mediatech, Manassas, VA, USA) at 37°C, 10% CO_2_). All experiments were performed utilizing charcoal-stripped serum. Cells were induced with 500 ng/ml doxycycline (Dox; Clontech) to express equivalent levels of AR and treated with 10 nM DHT (Sigma Aldrich). Stable REGγ transformants were selected with puromycin (2 μg/ml; Invitrogen, Inc.). Proteasome inhibitor epoxomycin (Sigma Aldrich) was used at 500 nM concentration.

### Plasmids and Site Directed Mutagenesis

FLAG-REGγ pcDNA3.1+plasmid (provided by Dr. Martin Rechsteiner, University of Utah). Site-directed mutagenesis (Quick Change II XL, Stratagene) was performed to mutate FLAG- REGγ into both REGγP245Y and REGγK188E. Mutations were confirmed by sequence analysis.

### REGγ PC12 Cell Lines

Stable transfections of Tet-On PC12 ARQ112 and ARQ10 cells were performed utilizing a calcium phosphate transfection protocol. FLAG-REGγ or FLAG-REGγP245Y plasmids were co-transfected with puromycin pBABE plasmid (kindly provided by Dr. Karen Knudsen, Thomas Jefferson University) at a 4:1 ratio, respectively. Single colonies were isolated, expanded, and screened for overexpressed REGγ using PSME3 antibody (BD Transduction Laboratories). Cells were maintained as stated above and media was supplemented with puromycin.

### Western Blot Analysis

PC12 cells and dissociated spinal cord cultures were lysed in 0.5% Triton-0.5% DOC lysis buffer and sonicated using a Branson cup sonifier. A DC protein assay (Bio Rad, Inc.) was performed to determine protein concentration. Protein lysates were electrophoresed by SDS-PAGE and transferred to a 0.45 μm PVDF (Immobilon-P) membrane. Western hybridization was performed using the following antibodies: [(AR(H280), AR(N20), AR(441) Santa Cruz Biotechnology), GAPDH (Fitzgerald Industries International)] (1:1000), [MDM2(SMP14); γ-tubulin (1:10,000) (Sigma Aldrich); REGγ (PSME3)] (1:2000) (BD Transduction Laboratories); FLAG (1:500) (Affinity BioReagents); SC35 (1:500) (Euromedex). Detection was carried out using ECL substrate (Pierce). Filter trap analysis was carried out as previously described (Bailey et al., 2002). Western analysis was performed using a standard protocol for antibody ARH280 (Santa Cruz Biotechnology).

### Immunofluorescence

PC12 cells and dissociated spinal cord cultures were fixed with 4% paraformaldehyde for 20 min, washed in PBS, permeabilized with 0.3% Triton X-100 for 15 min, blocked in 2% goat serum (Jackson ImmunoResearch, West Grove, PA, USA) for 20 min and incubated with primary antibody diluted in 1.5% goat serum for 1 h. Cells were washed in PBS and incubated with secondary antibody (FITC- or Texas Red-conjugated; Jackson ImmunoResearch, West Grove, PA, USA) for 30 min, washed with PBS, incubated in Hoechst (2 μg/ml), washed in PBS and mounted in Vectashield (Vector Laboratories, Burlingame, CA, USA). Fluorescence was visualized with a Leica (Leica Microsystems GmbH, Wetzlar, Germany) microscope and pictures were taken with a Leica camera and compiled with IP Lab software (BD Biosciences, Rockville, MD, USA). Antibodies utilized included the following: [AR(N20), AR(H280)] (1:100), p16(M-156) (1:50) (Santa Cruz Biotechnology); PSME3 (1:100) (BD Transduction Laboratories); FLAG (1:100) (Affinity BioReagents); SMI32 (1:1000) (Sternberger Monoclonal, Baltimore, MD, USA).

### Co-Immunoprecipitation Assay

PC12 cells were either treated with Dox (500 ng/ml) for 48 h and DHT (10 nM) in the presence or absence of epoxomycin (500 nM) for the last 12 h or with Dox (500 ng/ml) and DHT (10 nM) for 72 h with or without epoxomycin (500 nM) for the last 12 h. Controls consisted of PC12 ARQ112 cells inducibly expressing FLAG-REGγ or FLAG-REGγP245Y that were not treated with Dox or DHT, but treated with epoxomycin for 12 h. Cells were harvested in NP-40 lysis buffer (0.15 M NaCl, 50 mM NaF, 50 mM Tris-HCl (pH 7.5), 0.5% NP-40, and protease inhibitors), sonicated and protein concentration determined. Cell lysates (250 μg) were precleared with magnetic Protein G Dynabeads (Invitrogen) for 1 h at room temperature. Precleared lysates were incubated with AR(H280) antibody (0.5 μg) overnight at 4°C. Protein G Dynabeads were incubated with lysate/antibody for 30 min at room temperature, unbound lysate was removed and the beads were washed four times with NP-40 lysis buffer. Product was eluted with 2× Laemmli buffer and electrophoresed by SDS-PAGE, transferred to PVDF and Western analysis performed.

### Adenovirus Construction, Titration and Purification

FLAG-REGγ and FLAG-REGγK188E adenoviruses (replication incompetent (deltaE1/E3) human adenoviral type 5 genome) were constructed utilizing BD Adeno-X™ Expression System 1 (BD Biosciences). In brief, the pShuttle2 plasmid (BD Biosciences) and the FLAG- REGγ plasmids were digested with NheI and NotI restriction enzymes and then ligated to each other. The pShuttle2/FLAG-REGγ plasmid and BD Adeno-X Viral DNA (provided) were then cut with I-CeuI and PI-SceI restriction enzymes and ligated to each other. The resulting BD Adeno-X Viral plasmid containing FLAG-REGγ was sequenced to ensure proper ligation and then purified and digested with PacI restriction enzyme in order to linearize the plasmid. The PacI digested plasmids were transfected into low passage HEK293T cells and then freeze/thawed to release adenovirus. Adenoviruses were purified utilizing the Virabind™ Adenovirus Miniprep kit (Cell Biolabs, Inc., San Diego, CA, USA) and then titered using the QuickTiter™ Adenovirus Immunoassay Kit (Cell Biolabs, Inc., San Diego, CA, USA) according to published protocols.

### Adenovirus Overexpression Assay

PC12 ARQ112 cells were plated at 5 × 10^5^ cells/well in a 6-well dish and then infected with the following adenoviruses at multiplicity of infection (MOI) 100: FLAG-REGγ, FLAG-REGγK188E, LacZ. Cells were treated with 50 μM DHT and 500 ng/ml Dox for 72 h and then harvested for protein or immunostained as described above. Percent cells infected by FLAG-REGγ and FLAG-REGγKE were measured by calculating the number of FLAG-positive cells/total number of cells. LacZ viral infection was determined by performing an X-gal stain. Cells were counted and percent of cells with nuclear inclusions was calculated. Statistical analysis was carried out as described below.

### siRNA Assay

For REGγ knockdown studies: PC12 ARQ112 cell pellets (4 × 10^6^ cells) were resuspended in 100 μl of Nucleofector solution V (Amaxa®) (Lonza Cologne GmbH) with 50 pmol REGγsiRNA (ON-TARGETplus SMART pool rat PSME3), 50 pmol Non-Targeting siRNA (ON-TARGETplus Non-Targeting siRNA #1) (Dharmacon) or 2 μg GFP plasmid (Amaxa). Cells were electrophoresed using the Amaxa Nucleofector System (program U-029) and then replated in 6-well and 24-well dishes. At 72 h post nucleofection, cells were treated with Dox and DHT for 48 h. Cells were harvested at 120 h total and either lysed in Triton DOC lysis buffer and run on SDS-PAGE gels or immunostained. Percentage of siRNA protein knockdown was determined by densitometry. Cells were counted and percent of cells with nuclear inclusions was calculated. Statistical analysis was carried out using the Student’s *t*-test.

### Transient Transfection

PC12 ARQ112 cells were electroporated with the Amaxa® Nucleofector® System (Lonza Cologne GmbH) as described above with the following plasmids: FLAG-REGγ, FLAG- REGγP245Y, or GFP. Twenty-four hours post-transfection, cells were treated with Dox (500 ng/ml) and DHT (10 nM) for 72 h. Cells were then either harvested for biochemical analysis (Triton-DOC lysis buffer) or immunostained as described above.

### GST-TUBE (Tandem Ubiquitin Binding Entity) Assay

Cells were grown in 100 mm dishes and induced with Dox (500 ng/ml) for 60 h and treated with DHT (10 nM) and epoxomycin (500 nM) for the last 12 h. Cells were lysed on the dish in tandem ubiquitin binding entity (TUBE) lysis buffer (20 nM Na_2_HPO_4_, 20 nM NaH_2_PO_4_ (pH 7.2), 50 mM NaF, 5 mM tetra-sodium pyrophosphate, 10 nM β-glycerophosphate, 2 mM EDTA, 1 mM DTT, 1% NP-40) supplemented with fresh N-ethyl maleimide (NEM; 10 mM), and protease inhibitors and then 40 μl of GST-TUBE2 (Lifesensors, Inc., Malvern, PA, USA) was added. Cells were incubated on ice for 15 mins and then clarified (12,000 rpm for 10 min). A DC protein assay was performed to determine protein concentration. Glutathione (GST) affinity resin (GE Healthcare, Inc.; 100 μl) was equilibrated according to the provided protocol. GST resin was added to the cell lysate/TUBEs (2.5 mg protein) and incubated at 4°C for 4 h. GST-resin was collected by centrifugation (1000× *g*, 4°C) for 5 min, unbound sample was removed and resins washed three times with TBST. Product was eluted in 2× Laemmli buffer and loaded on a 10% SDS-PAGE gel for electrophoresis and transferred to 0.45 mM PVDF (Immobilon-P). Western hybridization was performed using the following antibodies: AR(441) (1:500) (Santa Cruz Biotechnology) and Ubiquitin (1:1000) (Dako).

### PC12 Cell Toxicity Assay

FLAG-REGγ-expressing PC12 cells, inducible for ARQ112 or ARQ10, were treated with 500 ng/ml Dox to express equivalent AR levels and then treated in the presence or absence of 10 nM DHT for 12 days. Cells were harvested and stained with trypan blue. Two hundred cells were counted per cell type/condition in triplicate. The percentage of trypan blue-positive cells was calculated and significance determined as described below.

### Construction of AAV Viruses

Adeno-associated viruses (AAV; type 1) were constructed by the following protocol: FLAG-RE REGγ pcDNA3.1 plasmids were cut with HindIII and XhoI restriction enzymes (Fisher Scientific) and the AAV-Cis plasmid (AM/CBA-pl-WPRE-bGH containing a chicken β-actin promoter and CMV enhancer) was cut with BamHI and XhoI restriction enzymes (Fisher Scientific); the two plasmids were ligated to form the FLAG-REGγ AAV-Cis plasmid. In addition, the pcDNA™6.2-GW/ EmGFP-miR plasmid, containing scrambled miRNA or miRNA sequences against SC35 (construction of these plasmids outlined below) was cut with XhoI and partially cut with DraI, while the AAV-Cis plasmid was cut with EcoRI and EcoRV. Both plasmids were treated with Klenow to blunt the ends and ligated together to form the scrambled AAV-, SC35 miRNA #24 AAV-, SC35 miRNA #27 AAV-Cis plasmids. Low passage HEK293T cells were plated and transfected with the FLAG-REGγ Cis, Trans (H21), and Adenovirus-associated Helper (δ6) plasmids using calcium phosphate. At three–5 days post-transfection, cells were harvested, lysed in PBS, freeze/thawed three times in order to obtain AAV viral stocks and then filtered. The volume of virus (HEK293T cell lysate) to achieve maximum AAV infection of motor neurons dissociated spinal cord cultures was determined by immunostaining for the protein of interest 5 days post-infection.

### Dissociated Spinal Cord Cultures

Dissociated spinal cord cultures were established according to published literature (Roy et al., 1998; Montie et al., 2009). Spinal cord cultures derived from transgenic ARQ112 mice were dissected on ice from 13.5-day-old embryos. Genotyping was carried out from a tail biopsy. Transgenic ARQ112 spinal cords were pooled, plated for culture and incubated for 3 weeks in conditioned media [MEM, 3% charcoal-stripped horse serum, 35 mM NaHCO_3_, 0.5% dextrose, 1% N3, 10 nM 2.5 S NGF (added after conditioning)] from 13.5-day-old non-transgenic brain. Motor neurons were identified in these mixed spinal cord cultures by staining with neurofilament heavy chain (SMI32) antibody and morphology. Three weeks after culture initiation, motor neurons were infected with the following AAV-viruses: EGFP, FLAG-REGγ, FLAG-REGγKE, FLAG-REGγP245Y, scrambled miRNA, or miRNAs against SC35 (#24 and #27). Five days following infection, motor neurons were treated with EtOH or 10 μM DHT for an additional 7 days. The work described here was carried out in strict accordance with the recommendations of The Guide for the Care and Use of Laboratory Animals of the National Institutes of Health, with approval by the Institutional Animal Care and Use Committee of Thomas Jefferson University.

### Creation of PC12 Cell Lines with Knockdown of SC35

Two pre-designed oligos (forward and reverse for each, Mmi519524 and Mmi519527) containing miRNA sequences against SC35, as well as scrambled, control sequences, were purchased from Invitrogen, annealed and cloned into pcDNA™6.2-GW/ EmGFP-miR (BLOCK-iT™), containing a blasticidin resistance gene, according to the manufacturer’s recommendations. Inducible PC12 cells (ARQ112) were stably transfected with these plasmids. Twenty-four hours post-transfection, cells were selected with (15 ng/mL) blasticidin. After 2 weeks in culture, 100% of the cells expressed EmGFP. Cells were treated with EtOH or 10 nM DHT for 12 days. At the end of the treatment, cells were harvested and stained for trypan blue. Two hundred cells were counted per cell type/condition in triplicate. The percentage of trypan blue-positive cells was calculated and significance was determined as described below.

### Statistical Analysis

Statistical analysis was carried out using either SigmaStat or Excel. For single factor analysis of variance (ANOVA), *post hoc* Bonferroni-corrected Student’s *t*-test was used to determine statistically significant differences. For two factor ANOVA, *post hoc* Tukey HSD was used to determine statistically significant differences. For single sample analysis with two conditions, Student’s *t*-test was used after determining sample variance with the F statistic.

## Results

### REGγ Is Present in ARQ112 Nuclear Inclusions

**Figure 1:**
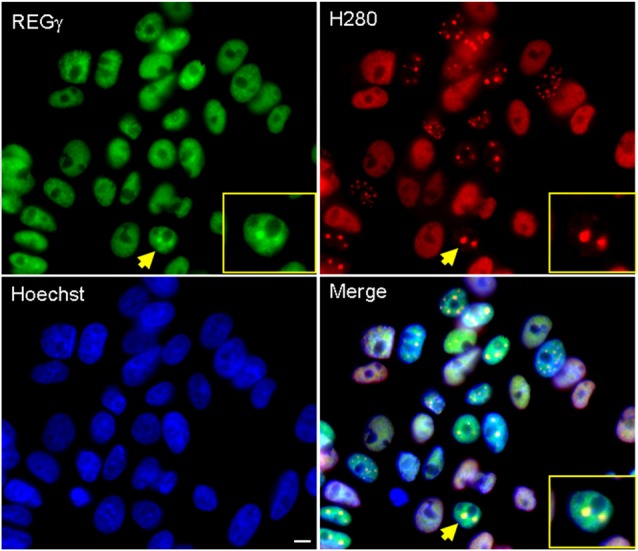
**REGγ colocalizes with ARQ112 in inclusion bodies in PC12 cells.** Immunofluorescence of PC12 ARQ112 cells induced with doxycycline (Dox) and treated with dihydrotestosterone (DHT) for 48 h. Cells were stained with androgen receptor (AR; H280) and REGγ antibodies. Hoechst staining was carried out to detect nuclei. Arrow indicates a cell positive for AR nuclear inclusions that co-localize with REGγ; this cell is magnified in the inset. Scale bar represents 5 μm.

Numerous studies have demonstrated that components of the UPS system are present within nuclear inclusions in cell and animal models of SBMA and tissue of affected SBMA patients (Li et al., 1998; Stenoien et al., 1999; Abel et al., 2001; Walcott and Merry, 2002); we therefore wished to determine whether REGγ co-localizes with hormone-induced ARQ112 nuclear inclusions in this cell model of SBMA. We observed REGγ both in a diffuse nuclear distribution and co-localized with a portion of AR nuclear inclusions (Figure 1).

### Both REGγ and REGγK188E Isoforms Cause a Substantial Increase in the Frequency of Cells Containing ARQ112 Nuclear Inclusion Bodies

**Figure 2:**
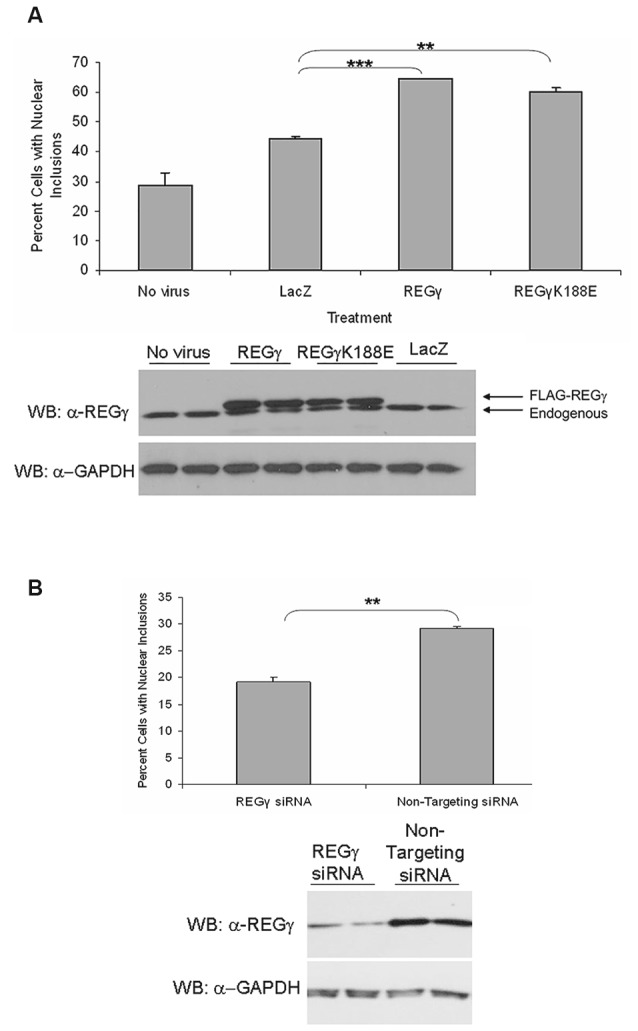
**Overexpression of REGγ and REGγK188E significantly increases the percentage of cells with ARQ112 nuclear inclusion bodies whereas decreased expression reduces this percentage. (A)** PC12 cells expressing ARQ112 were infected with adenovirus expressing FLAG-REGγ, FLAG-REGγK188E or LacZ at multiplicity of infection 100 (MOI 100). Cells were then treated with Dox to induce AR in the presence of DHT for 72 h. Cells were immunostained with antibodies to the AR (N20), to FLAG for infectivity and stained with Hoechst to reveal nuclei. The percentage of cells with nuclear inclusions was calculated. Single factor analysis of variance (ANOVA) with *post hoc* Bonferroni-corrected *t*-test was performed. **p < 0.01; ***p < 0.001. The above PC12 ARQ112 cells were also harvested at 72 h and evaluated for REGγ protein levels via Western blot analysis. Note the overexpression of FLAG-REGγ compared to endogenous protein levels. **(B)** PC12 ARQ112 cells were transfected with siRNA against REGγ or with a non-targeting siRNA control for 72 h. Cells were then induced with Dox and treated with DHT for 48 h. Cells were harvested after 120 h to evaluate REGγ protein levels via Western blot analysis. Densitometry showed 77% knockdown of REGγ protein. Cells were immunostained at 120 h with AR(N20) antibody and stained with Hoechst. The percentage of cells with nuclear inclusions was calculated. Student’s *t*-test was performed. **p < 0.01.

The presence of REGγ in AR nuclear inclusions suggests that nuclear REGγ-capped proteasomes may play a role in the inefficient degradation of mutant AR. Moreover, it has been previously shown that REGγ is capable of activating only the T-L activity of the 20S proteasome core, thereby limiting the degradative capacity of nuclear 11S proteasomes (Realini et al., 1997). However, mutation of REGγ at lysine 188 to an acidic residue (K188E, K188D), as found in the cytoplasmic REG homologs REGα and β (Zhu et al., 2001), allows the activation of all three catalytic activities of the REGγ-capped 20S proteasome. We therefore sought to investigate whether overexpression of REGγ or REGγK188E would impact the aggregation of expanded AR. We predicted that REGγK188E might enhance the nuclear degradation of expanded full-length AR or expanded AR fragments due to its REGα/β-like activation capabilities and thereby decrease AR aggregation. The cell model that we used to test this hypothesis is a well characterized inducible PC12 cell model of SBMA, in which hormone treatment of cells expressing an *AR* gene with 112 CAG repeats (ARQ112) results in the formation of nuclear inclusions comprised of an amino-terminal AR fragment (Walcott and Merry, 2002; Heine et al., 2015), as observed in SBMA patient neurons (Li et al., 1998). Moreover, nuclear inclusions correlate well with a subset of biochemical AR aggregates detected by SDS-agarose gel electrophoresis (SDS-AGE; Berger et al., 2015; Heine et al., 2015). To test our hypothesis, adenoviruses were created to express FLAG-tagged REGγ, FLAG-REGγK188E or LacZ in PC12 ARQ112 cells. Overexpression of both FLAG-REGγ and FLAG-REGγK188E resulted in a substantial increase in the frequency of cells with ARQ112 nuclear inclusions, compared to the LacZ control (Figure 2A).

We next wanted to determine whether knockdown of REGγ would impact expanded AR aggregation. Knockdown of REGγ caused a significant decrease in ARQ112 inclusion bodies, but did not eliminate them (Figure 2B). Thus, not only did the overexpression of REGγ enhance AR aggregation, but the knockdown of endogenous REGγ decreased AR aggregation, suggesting a role for REGγ in the aggregation of mutant AR.

### The REGγ-Induced Increase in ARQ112 Inclusion Bodies Does Not Require its Binding to the 20S Proteasome Core

**Figure 3:**
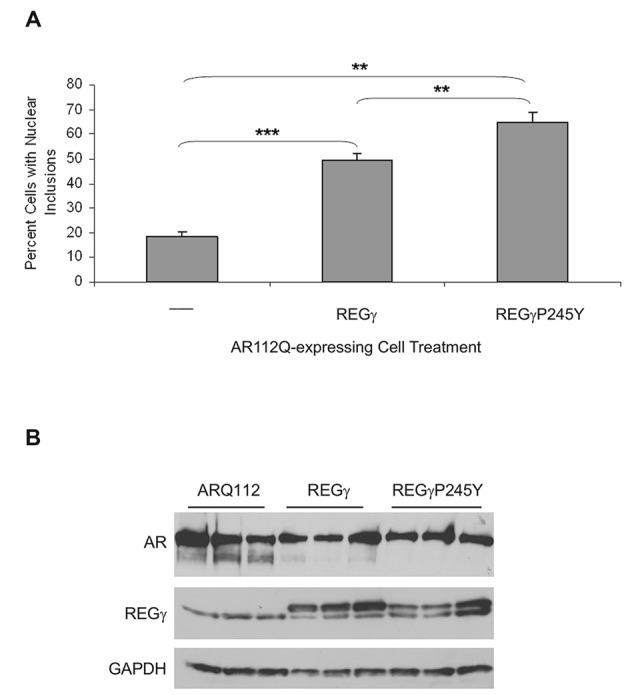
**Overexpression of REGγP245Y mutant significantly increases the percentage of cells with ARQ112 nuclear inclusion bodies.** PC12 ARQ112 cells that stably overexpress either REGγ or REGγP245Y were induced with Dox and treated with DHT for 72 h. **(A)** Cells were immunostained with AR(H280) and stained with Hoechst. The number of cells containing nuclear inclusions was determined and their percentage calculated. Single factor ANOVA with *post hoc* Bonferroni-corrected *t*-test was performed. **p < 0.01; ***p < 0.001. See Supplementary Figure S1 for additional analysis of AR aggregation. **(B)** Western blot analysis of same cell populations as in **(A)** reveals lack of effect of REGγ or REGγP245Y overexpression on monomeric AR levels.

One possible explanation for these results is that REGγ heptameric rings compete with the 19S proteasomal activator, thereby decreasing AR degradation and enhancing its aggregation. Therefore, given that REGγ has functions that are both proteasome binding-dependent and proteasome binding-independent, we asked whether proteasome binding by REGγ is required for these effects. Proline 245 was previously shown to be required for the binding of REGγ to the 20S proteasome α rings (Zhang et al., 1998; Zhang and Zhang, 2008). To determine whether interaction of REGγ with the 20S proteasome core is required for the observed REGγ effects on ARQ112 aggregation, we created PC12 ARQ112 cells that stably express either REGγ or the proteasome-binding mutant REGγP245Y. We found that stable overexpression of both REGγ and REGγP245Y significantly increased ARQ112 aggregation (Figure 3A, Supplementary Figure S1), indicating that REGγ increases expanded AR aggregation in a 20S proteasome-binding-independent manner. Moreover, mutant REGγP245Y had a slightly greater effect on increasing ARQ112 aggregation, compared to wild-type REGγ, possibly due to its limited non-proteasome binding role. The same aggregation results were observed upon transient overexpression of both constructs (data not shown). Neither overexpression of REGγ or of REGγP245Y altered the steady state levels of ARQ112 (Figure 3B).

### REGγ Interacts with ARQ112

**Figure 4:**
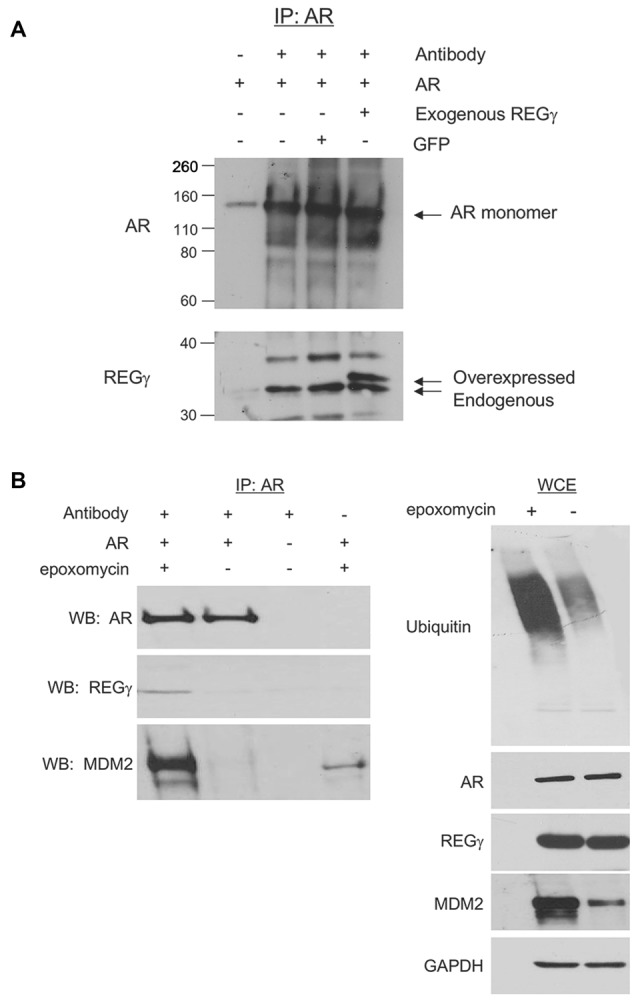
**AR interacts with REGγ. (A)** PC12 ARQ112 cells were transiently transfected with FLAG-REGγ or GFP and then induced with Dox and treated with DHT for 72 h. Lysates were immunoprecipitated with AR(H280) antibody, separated by SDS-PAGE and protein complexes detected by Western analysis using AR(318) and REGγ antibodies. Included controls are no antibody and no transfection. **(B)** PC12 ARQ112 cells were induced with Dox and treated with DHT for 48 h and then treated with or without epoxomycin for an additional 12 h. (Left) AR was immunoprecipitated with AR(H280) antibody. Included controls have no AR expression or no antibody in the IP. Western blot analysis was carried out with AR(318), REGγ and MDM2 antibodies to evaluate protein complexes. (Right) Whole cell extracts (WCE) used for IP were evaluated for respective protein levels by Western blot analysis. GAPDH was employed as a loading control.

With the observation that REGγ promotes AR aggregation in a proteasome binding-independent manner, we sought to determine the mechanism for this effect. We found that full-length ARQ112 co-immunoprecipitated with both endogenous and exogenous (overexpressed) REGγ (Figures 4A,B). Moreover, treatment of cells with the proteasome inhibitor epoxomicin enhanced the interaction of REGγ with ARQ112 (Figure 4B), without changing the steady-state levels of REGγ or AR (Figure 4B, right). We also found that REGγP245Y co-immunoprecipitated with ARQ112 (Figure 7), indicating that REGγ can interact with ARQ112 independently of REGγ association with the proteasome. We did not detect an interaction of REGγ with unexpanded ARQ10 (data not shown), suggesting that REGγ has a limited, if any, role in normal AR metabolism.

**Figure 5:**
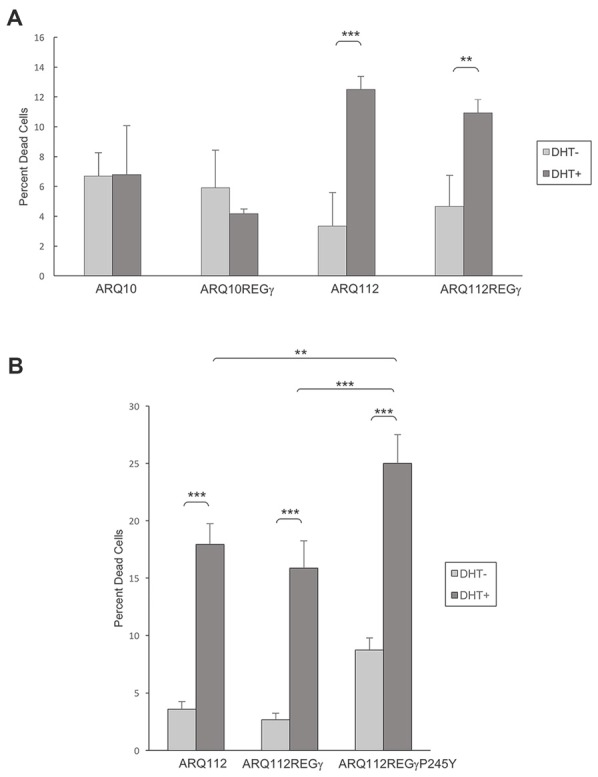
**Overexpression of REGγ and mutant REGγP245Y fail to protect PC12 ARQ112 cells from DHT-dependent death. (A,B)** ARQ10 and ARQ112 cells with and without stable expression of REGγ or REGγP245Y were cultured in the presence or absence of DHT for 12 days. Cells were stained with trypan blue, 200 cells were counted in triplicate, and the percentage of dead cells (trypan blue-positive) was calculated. Data are representative of more than three independent experiments. Statistical analysis was carried out using two-factor ANOVA with *post hoc* Tukey HSD for individual comparisons. **p < 0.01; ***p < 0.001.

**Figure 6:**
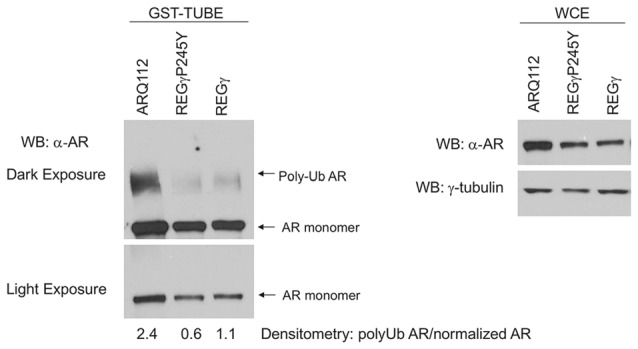
**Overexpression of REGγ and REGγP245Y decreases the polyubiquitination state of ARQ112.** PC12 ARQ112 cells were induced with Dox for 48 h, and then treated for an additional 12 h with Dox, DHT and epoxomycin. Cells were harvested in tandem ubiquitin binding entity (TUBE) cell lysis buffer and treated with GST-TUBE2. TUBE assay protocol was carried out and the eluate was evaluated by Western analysis using anti-AR antibody AR(441) to detect polyubiquitinated ARQ112 (left). Densitometry was performed and the ratio of polyubiquitinated AR to WCE normalized AR monomer (right) was calculated.

**Figure 7:**
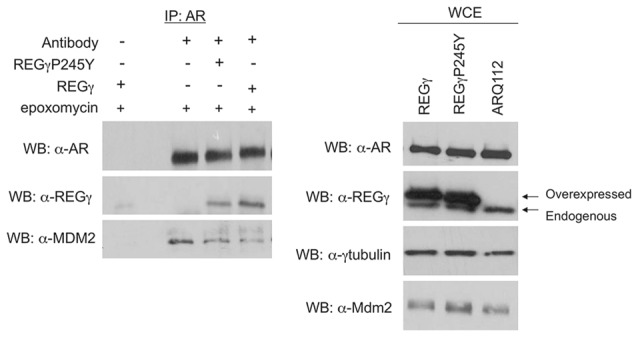
**Overexpression of REGγ decreases ARQ112 interaction with MDM2.** PC12 ARQ112 cells stably overexpressing REGγ or REGγP245Y were induced with Dox for 48 h and treated with DHT and epoxomycin for the last 15 h. Protein lysates were harvested at 48 h and then AR was co-immunoprecipitated with AR(H280) antibody (left). Protein complexes were evaluated via Western blot analysis using AR(441), REGγ and MDM2 antibodies. (Right) Western blots of whole cell lysates (WCE) used for IP were probed with antibodies detecting REGγ, AR (AR(318)), MDM2 and γ-tubulin.

### Both REGγ and REGγP245Y Fail to Protect SBMA PC12 Cells from DHT-Dependent Death

Utilizing PC12-AR-expressing cell lines that we engineered to stably overexpress REGγ or REGγP245Y, we examined whether overexpression of both isoforms modulated DHT-dependent cell death. We found that ARQ112 cells stably overexpressing either REGγ or REGγP245Y were not protected from DHT-induced AR toxicity (Figures 5A,B), and neither of the REGγ isoforms caused death in cells expressing unexpanded ARQ10 (Figure 5A, data not shown). Moreover, REGγP245Y overexpression resulted in slightly increased toxicity (Figure 5B), as observed with its effect on AR aggregation.

### Overexpression of REGγ and REGγP245Y Decreases Polyubiquitination of ARQ112

REGγ has been shown to promote the polyubiquitination of p53 in a proteasome binding-independent manner (Zhang and Zhang, 2008). We therefore wanted to evaluate whether REGγ overexpression modified the polyubiquitination state of ARQ112. We observed that the overexpression of both REGγ and REGγP245Y significantly decreased the polyubiquitination state of ARQ112 (Figure 6). While the total amount of ubiquitin pulled down from the lysates was equivalent across cell lines (data not shown), the amount of polyubiquitinated ARQ112 was reduced in cells expressing either REGγ or REGγP245Y. Therefore, REGγ negatively impacts ARQ112 polyubiquitination. The apparent decrease in ARQ112 polyubiquitination might be explained by increased ARQ112 aggregation and consequent insolubility. However, evaluation of ARQ112 polyubiquitination was carried out under conditions that minimized AR aggregation; indeed, no AR-containing nuclear inclusions were observed in the short time of DHT treatment (data not shown). Moreover, we evaluated AR solubility using ultracentrifugation and Western blot analysis; no increase in ARQ112 sedimentation was observed under these conditions. Consistent with its lack of interaction with ARQ10, REGγ had no effect on the polyubiquitination state of ARQ10 (data not shown).

We next wanted to evaluate the mechanism by which REGγ decreases the polyubiquitination of ARQ112. A recent study showed that REGγ facilitates the interaction of the E3 ubiquitin ligase MDM2 with p53, thus promoting the polyubiquitin-dependent degradation of p53 (Zhang and Zhang, 2008). MDM2 is a known E3 ubiquitin ligase for the AR (Lin et al., 2002); therefore, we hypothesized that REGγ might prevent the interaction of ARQ112 with MDM2, resulting in reduced polyubiquitination of the receptor. We observed MDM2 co-immunoprecipitation with polyQ-expanded AR only in the presence of the proteasome inhibitor epoxomycin (Figure 4B), which substantially increased the otherwise low levels of MDM2 in these cells (Figure 4B, right). Consistent with our finding that REGγ overexpression decreased AR polyubiquitination, we found that REGγ overexpression also decreased the interaction of ARQ112 with MDM2 (Figure 7), suggesting that REGγ may decrease ARQ112 polyubiquitination by reducing the interaction of ARQ112 with the ubiquitin ligase MDM2.

### Overexpression of REGγ and REGγK188E Protects Transgenic SBMA Motor Neurons from DHT-Dependent Cell Death

**Figure 8:**
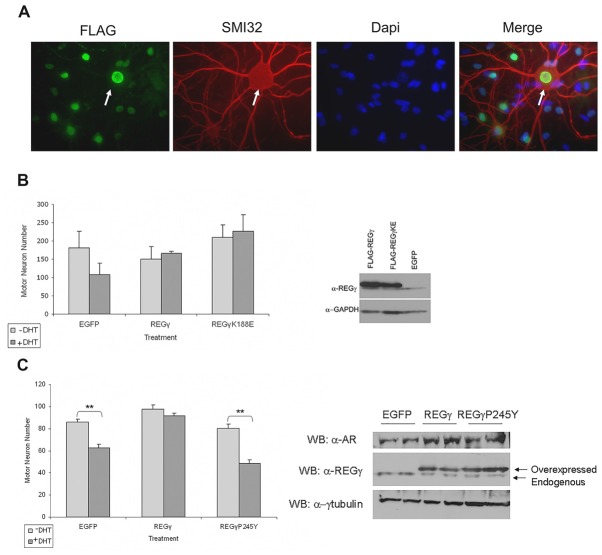
**REGγ and REGγK188E overexpression rescued motor neurons from DHT-dependent death, while REGγP245Y overexpression did not. (A)** Dissociated spinal cord cultures from ARQ112 transgenic mouse embryos were cultured for 3 weeks. Cultures were infected with adeno-associated virus (AAV) expressing REGγ, REGγK188E or EGFP for 5 days and then treated with or without DHT for 7 days. Cultures were immunostained with FLAG antibody to detect FLAG-REGγ and FLAG-REGγK188E infected cells, with an antibody against neurofilament heavy chain (SMI32) to reveal motor neurons and stained with Hoechst for nuclei. Arrow indicates a motor neuron infected with nuclear FLAG-REGγ. **(B)** Left, motor neurons from immunostained cultures in **(A)** were counted in 10 random fields (2 wells per condition). Two factor ANOVA with *post hoc* TUKEY HSD was performed. The difference in the number of motor neurons between the +DHT and –DHT conditions for EGFP was not statistically significant (*p* = 0.05). Note, however, the complete lack of cell death in the REGγ- and REGγK188E- expressing cultures. Right, protein lysates from the above cultures were evaluated by Western blot for REGγ and GAPDH (loading control). **(C)** Left, motor neurons were infected with EGFP, FLAG-REGγ and FLAG-REGγP245Y expressing AAV for 5 days and treated with or without DHT for 7 days. Cultures were immunostained with SMI32 antibody and motor neurons in 10 random fields in each of three wells per condition were counted. Two factor ANOVA with *post hoc* TUKEY HSD was performed. **p < 0.01. Right, protein lysates were evaluated by Western blot with AR(H280), REGγ and γ-tubulin antibodies.

Our finding that REGγ overexpression in PC12 cells significantly increased ARQ112 aggregation led us to ask whether REGγ overexpression would impact SBMA motor neuron survival. Transgenic ARQ112 motor neurons undergo significant hormone-dependent death (Montie et al., 2009, 2011; Orr et al., 2010; Heine et al., 2015; Zboray et al., 2015). We hypothesized, based on our observations in PC12 cells, that overexpression of REGγ and REGγK188E might enhance motor neuron death after exposure to hormone. To test this hypothesis, we infected dissociated spinal cord cultures with AAV that expressed FLAG-REGγ isoforms. A high percentage of motor neurons were infected (Figure 8A), and significant overexpression of REGγ isoforms was observed (Figure 8B, right). In contrast to our observations in PC12 cells, however, we found that overexpression of either REGγ or REGγK188E not only failed to enhance DHT-dependent toxicity, but rather rescued ARQ112 motor neurons from DHT-induced toxicity (Figure 8B, left). Moreover, REGγ protected ARQ112 motor neurons from DHT-dependent toxicity in a proteasome binding-dependent manner, as overexpression of the proteasome non-binding mutant REGγP245Y did not rescue motor neuron viability (Figure 8C). This suggests that the effect of REGγ on motor neuron survival depends on a REGγ proteasome binding (11S)-dependent mechanism.

We next determined whether REGγ overexpression rescued motor neurons from DHT-dependent cell death by altering levels of the mutant polyQ-expanded AR. Western analysis revealed that REGγ overexpression did not alter AR monomer levels (Figure 8C, right), suggesting that REGγ does not promote motor neuron survival by enhancing ARQ112 degradation. The effect of REGγ on AR aggregation and inclusion formation in cultured motor neurons could not be evaluated, as inclusions do not form in these neurons during the time course of the toxicity assays performed here (Heine et al., 2015).

### REGγ Effects May be Mediated by the Splicing Factor SC35

**Figure 9:**
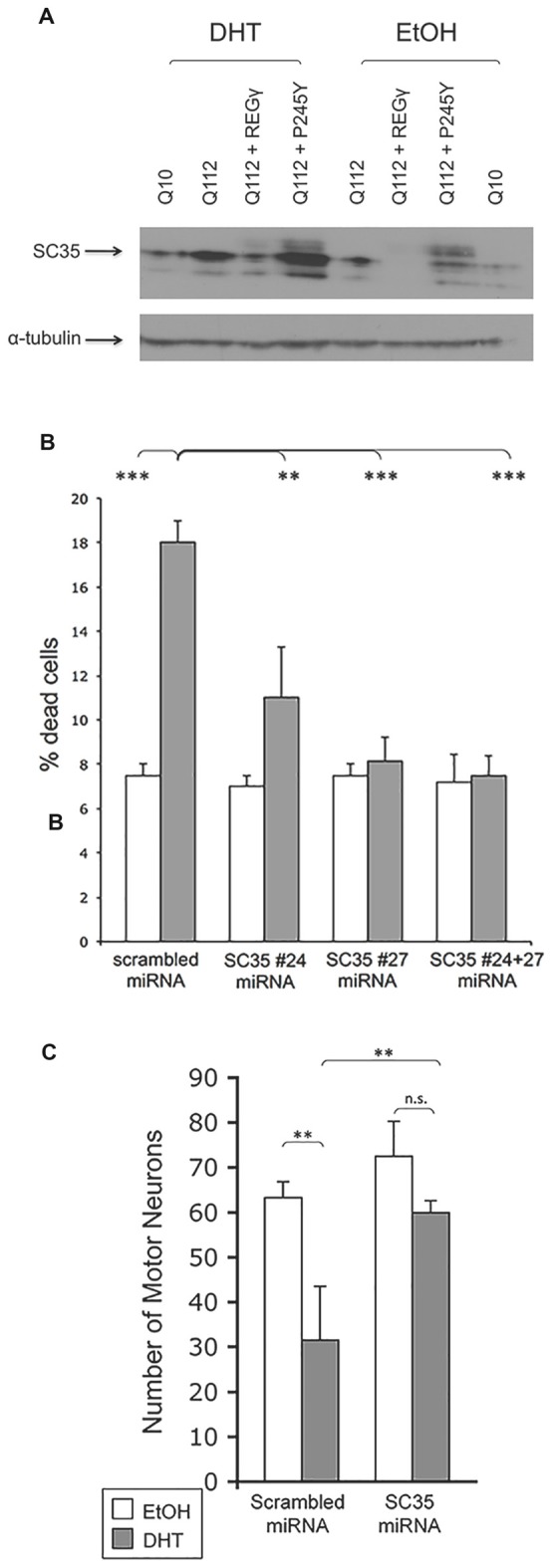
**REG– effects may be mediated by the splicing factor SC35. (A)** SC35 levels are increased in the presence of DHT in PC12 cells expressing ARQ112. REGγ overexpression ameliorates enhanced SC35 levels in a manner dependent upon its ability to bind the proteasome. PC12 cells expressing ARQ10, ARQ112 and ARQ112 + REGγ and ARQ112 + REGγ (P245Y) were treated with EtOH or DHT for 48 h, harvested and Western analysis was performed to determine SC35 levels. **(B)** Knockdown of SC35 rescues PC12 cells expressing ARQ112 from DHT-dependent death. PC12 cells expressing ARQ112 were stably (bulk) transfected with plasmids containing scrambled miRNA or two miRNAs against SC25 (#24 and #27) and treated with EtOH or DHT for 12 days, after which cell death was assessed by trypan blue exclusion. SC35 #24 miRNA = 21% knockdown, SC35 #27 miRNA = 30% knockdown, SC35 #24 + 27 miRNA = 40% knockdown. Two factor ANOVA with *post hoc* TUKEY HSD was performed. **p < 0.01; ***p < 0.001. See Supplementary Figure S2 for representative images used for SC35 quantification.** (C)** Knockdown of SC35 rescues primary motor neurons expressing ARQ112 from DHT dependent death. Dissociated spinal cord cultures from ARQ112 transgenic mouse embryos were cultured for 3 weeks. Cultures were infected with AAV expressing scrambled miRNA or miRNA against SC35 (#27) for 5 days and then treated with EtOH or 10μM DHT for 7 days. Cultures were immunostained with an antibody against neurofilament heavy chain (SMI32) to reveal motor neurons and stained with Hoechst for nuclei. Green fluorescence revealed the motor neurons that were infected with AAV, as the miRNA constructs are tagged with EmGFP. Infectivity, scrambled = 75%, miRNA against SC35 (#27) = 77%. Two factor ANOVA with *post hoc* TUKEY HSD was performed. **p < 0.01; n.s., not statistically significant.

The observation that the neuroprotection conferred by REGγ in SBMA mouse motor neurons requires its ability to bind the 20S proteasome core prompted us to evaluate potential REGγ substrates that mediate its effects. Several REGγ-activated proteasomal substrates have been identified. These include the CDK inhibitors p21^WAF/CIP1^, p16^INK4a^ and p19^ARF^ (Chen et al., 2007) the transcriptional co-activator SRC-3 (Li et al., 2006) and the E3 ligase SMURF1 (Nie et al., 2010). We evaluated levels of p16^INK4a^ in motor neurons in the presence of overexpressed REGγ but found no change in its levels (unpublished data). Another potential substrate of REGγ-capped proteasomes is the nuclear splicing factor SC35. REGγ-capped proteasomes colocalize with SC35-containing nuclear speckles (Baldin et al., 2008). Moreover, knockdown of REGγ leads to altered SC35 distribution. To evaluate the potential role of SC35 in REGγ-induced protection of SBMA motor neurons, we first determined the effect of ARQ112 expression on SC35 levels, using the PC12 cell model described above. SC35 is transcriptionally regulated by E2F1 (Merdzhanova et al., 2008) and is consequently present at much higher (and detectable) levels in cycling PC12 cells than in primary motor neurons (unpublished data). Expression of polyQ-expanded AR resulted in increased expression of SC35, which was further increased by DHT treatment (Figure 9A). Moreover, overexpression of REGγ, but not the proteasome binding-mutant REGγ-P245Y, resulted in the reduction in SC35 levels (Figure 9A).

To determine the extent to which decreased SC35 levels contributed to REGγ neuroprotection, we knocked down SC35, in the absence of overexpressed REGγ and determined the effect on cellular viability. ARQ112-expressing PC12 cells were stably transfected with either a plasmid expressing scrambled miRNA or a plasmid expressing miRNA against SC35 and placed under blasticidin selection for 2 weeks; after 2 weeks, all cells were confirmed to express EmGFP, which is contained on the same expression plasmid. Cells were then treated with EtOH or DHT and viability assessed after 12 days. Reducing SC35 expression provided complete protection against DHT-dependent death (Figure 9B). To determine if knockdown of SC35 would also prevent DHT-dependent ARQ112 toxicity in primary motor neurons derived from SBMA transgenic mice, we infected dissociated spinal cord cultures from ARQ112-expressing mice with AAV expressing either a scrambled miRNA or a miRNA against SC35 and evaluated the effect on DHT-dependent AR toxicity. Knockdown of SC35 in primary SBMA motor neurons also substantially reduced DHT-dependent AR toxicity (Figure 9C).

## Discussion

The role of nuclear degradative processes in polyQ diseases has garnered much interest since the discovery of nuclear inclusions. In SBMA, in particular, in which the site of pathogenesis is the nucleus (Takeyama et al., 2002; Montie et al., 2009; Nedelsky et al., 2010), identifying the role of nuclear proteases in the inefficient degradation and aggregation of the mutant AR may be critical to understanding the disease process. PolyQ-expanded AR displays altered nuclear metabolism; while it is able to transactivate target genes, at some point in its nuclear metabolism, it misfolds and forms nuclear inclusions that consist of amino-terminal fragments (Li et al., 1998; Walcott and Merry, 2002; Li M. et al., 2007; Heine et al., 2015). Nuclear inclusions also contain molecular chaperones, ubiquitin, and proteasomal subunits such as the 20S proteasome core and REGγ, a nuclear proteasomal activator (Li et al., 1998; Stenoien et al., 1999; Abel et al., 2001; Bailey et al., 2002). This finding suggested that REGγ might play a role in mutant AR metabolism and led us to further explore the role of REGγ in SBMA pathogenesis.

Our entrée into this question focused on the restricted proteasomal catalytic activity that is induced by nuclear REGγ (Realini et al., 1997), and we hypothesized that this feature contributes to the inefficient proteasomal degradation of nuclear AR. However, we found that the overexpression of both REGγ and REGγK188E not only increased, rather than decreased, ARQ112 aggregation in PC12 cells, but that this effect of REGγ on aggregation is independent of the manner in which it activates the proteasomal core. Consistent with this observation, REGγ (P245Y), which is unable to bind to the 20S proteasome core, also increased ARQ112 aggregation, implicating a mechanism that is independent of REGγ’s role as an 11S proteasomal activator.

Our studies here identified one proteasome binding-independent mechanism of REGγ, which may impact ARQ112 aggregation, as its inhibition of MDM2 binding to the mutant AR. We focused on MDM2 in our studies, as MDM2 can polyubiquitinate normal AR (Lin et al., 2002) and because REGγ had been shown to modify MDM2 binding to another MDM2 substrate, p53 (Zhang and Zhang, 2008). We found that MDM2 interacts with expanded ARQ112 and that REGγ overexpression decreased this interaction; preliminary findings also suggest that it decreases ARQ112 interaction with E6AP, another known AR E3 ligase (data not shown). The fact that multiple E3 ligases may be impacted by REGγ is notable, as E3 ubiquitin ligases may act redundantly to promote the degradation of poly-Q expanded proteins such as AR and ataxin-3 (Morishima et al., 2008). Further studies will be needed to determine if the decrease in ARQ112 interaction with E3 ligases is responsible for the decrease in ARQ112 polyubiquitination. It is also possible that REGγ may promote the degradation of E3 ubiquitin ligases involved in AR degradation, since a recent study showed that REGγ is capable of interacting with and promoting the degradation of the E3 ubiquitin ligase Smurf1 (Nie et al., 2010); of note, however, no effect of REGγ on MDM2 levels was observed (Figure 7, right).

Whether and how such a decrease in AR polyubiquitination plays a role in its increased aggregation is unknown. Our finding that REGγ decreases expanded AR polyubiquitination predicts that it would increase expanded AR half-life. Although we found no effect of REGγ on steady-state levels of monomeric AR (Figures 3B, 7) or on AR half-life (data not shown; evaluated prior to its aggregation), it may be that REGγ interacts with a subset of AR protein, impacting its degradation and thus overall aggregation and toxicity. Identifying such an AR subpopulation is the subject of ongoing studies.

To study the effect of REGγ on toxicity in motor neurons, we utilized transgenic motor neurons derived from a mouse model of SBMA (Mojsilovic-Petrovic et al., 2009; Montie et al., 2009, 2011; Orr et al., 2010; Heine et al., 2015; Zboray et al., 2015). These mice develop the slowly progressive motor deficits and pathology seen in SBMA patients (Chevalier-Larsen et al., 2004). Our finding that REGγ, REGγK188E, and REGγP245Y increased mutant AR aggregation in PC12 cells predicted that the increased expression of REGγ isoforms would promote DHT-dependent motor neuron death. To our surprise, we found that both REGγ and REGγK188E rescued motor neurons from DHT-dependent death. Moreover, this rescue occurred in a proteasome binding-dependent manner, as REGγP245Y failed to protect motor neurons from AR toxicity.

REGγ has been previously shown to increase the viability of mutant huntingtin (Htt)-expressing striatal neurons after exposure to cell stressors (Seo et al., 2007). Thus, REGγ can enhance the survival of distinct neuronal populations in two models of polyQ disease. How REGγ promotes neuronal survival is unknown. We found that REGγ does not promote motor neuron survival by enhancing ARQ112 degradation. Instead, REGγ may increase the destruction of other substrates involved in the pathogenesis of polyQ disease, leading to enhanced neuronal viability. We found that SC35, which is altered by REGγ knockdown and is present in nuclear speckles along with active REGγ-capped 11S proteasomes (Baldin et al., 2008), is increased upon DHT-treatment of polyQ-expanded AR-expressing PC12 cells and reduced by REGγ, but not REGγ-P245Y, expression. Moreover, reducing SC35 levels in both PC12 cells and motor neurons rescued both cell types from DHT-dependent toxicity.

Our studies of REGγ on ARQ112 toxicity revealed distinct effects on PC12 cells and motor neurons. We propose that REGγ is involved in competing proteasome binding-dependent and -independent pathways. The proteasome binding-dependent REGγ pathway is predominant in motor neurons and is neuroprotective. In contrast, the proteasome binding-independent REGγ pathway predominates in PC12 cells and promotes AR aggregation and toxicity. Consistent with this view, REGγP245Y overexpression promoted AR toxicity in PC12 cells, while it failed to protect motor neurons from DHT-dependent toxicity. The distinct effects of REGγ in PC12 cells and transgenic motor neurons are in contrast with the common neuroprotective effect of SC35 knockdown in both cell types. It may be that the interaction of ARQ112 with REGγ reduces the nuclear levels of REGγ-capped 11S proteasomes. Increasing levels of REGγ in motor neurons may be sufficient to restore 11S proteasome degradative capacity, thus reducing the levels of key 11S proteasome substrates, including SC35. However, the higher levels of SC35 in cycling PC12 cells may not be sufficiently reduced by REGγ overexpression, shifting the balance of REGγ effects to one of proteasome binding-independent interaction with the mutant AR.

A previous study tested the hypothesis that REGγ knockdown would improve phenotype in HD R6/2 mice. However, the knockout of REGγ had no effect on HD disease progression or formation of nuclear inclusions (Bett et al., 2006). While the site of SBMA pathogenesis is clearly restricted to the nucleus, in HD, pathogenic mechanisms likely involve both the nucleus and cytoplasm. An effect of REGγ, a nuclear protein, might be diluted by a cytoplasmic disease mechanism. Moreover, based on our findings that REGγ protects motor neurons expressing mutant AR, we predict that the knockout of REGγ would exacerbate disease; whether this could be observed in the severe R6/2 model of HD is unknown. Our findings of REGγ protection of motor neurons predict that overexpressing REGγ rather than knocking it down, would improve polyQ expansion disease phenotype, not only for SBMA but for other polyQ repeat diseases as well.

The observed rescue of motor neurons from DHT toxicity by REGγ, in a proteasome binding-dependent manner, predicts that the composition of nuclear proteasomes was altered by its overexpression. Native PAGE analysis of PC12 nuclear fractions failed to reveal such a difference (data not shown). However, given the limited abundance of nuclear proteasomes, it is likely that native PAGE lacks the necessary sensitivity to reveal such changes. Also, REGs are in rapid equilibrium with proteasomes (Welk et al., 2016), making such measurements of proteasomal composition difficult to determine with accuracy. Nonetheless, we predict that REGγ overexpression increases 11S-containing proteasomes, thus enhancing the degradation of 11S proteasomal substrates involved in motor neuron death.

In conclusion, these studies indicate that REGγ is involved with polyQ-expanded AR metabolism through two distinct mechanisms. In its proteasome binding-independent role, REGγ decreases the interaction of ARQ112 with E3 ubiquitin ligases, leading to a reduction in mutant AR polyubiquitination and promoting its aggregation and toxicity in PC12 cells. By contrast, REGγ promotes motor neuron viability through a proteasome binding-dependent mechanism, likely involving increased levels of 11S proteasomes and leading to the degradation of 11S proteasomal substrates, potentially including SC35. These findings suggest a model in which mutant AR binding to REGγ in transgenic motor neurons reduces its activation of 11S proteasomes and leads to increased levels of 11S proteasomal substrates. We predict that therapies directed at enhancing the role of REGγ in 11S-proteasome degradation may prove beneficial in preventing toxicity in SBMA and other polyQ diseases. Additional studies are essential to fully understand the mechanism by which REGγ proteasome binding promotes motor neuron viability, as well as to further comprehend how the alternative roles of REGγ degradation affect nuclear metabolism in the context of neurodegenerative diseases.

## Author Contributions

JMY, HLM, ESC-L, MR and DEM designed the experiments. JMY, HLM, ESC-L, YL and LH performed the experiments. JMY, HLM, ESC-L, YL, LH and DEM analyzed the data. JMY and DEM wrote the manuscript; prepared the figures. All authors read, reviewed, edited and commented on the manuscript.

## Conflict of Interest Statement

The authors declare that the research was conducted in the absence of any commercial or financial relationships that could be construed as a potential conflict of interest.
