# Supplementary material for: The 11S Proteasomal Activator REGγ Impacts Polyglutamine-Expanded Androgen Receptor Aggregation and Motor Neuron Viability through Distinct Mechanisms
Source: Front Mol Neurosci. 2017 May 24;10:159. doi: 10.3389/fnmol.2017.00159 (PMC5442185; doi:10.3389/fnmol.2017.00159)
Supplement: Supplementary file 1 [file Presentation_1.pdf]

Supplemental Figure 1.

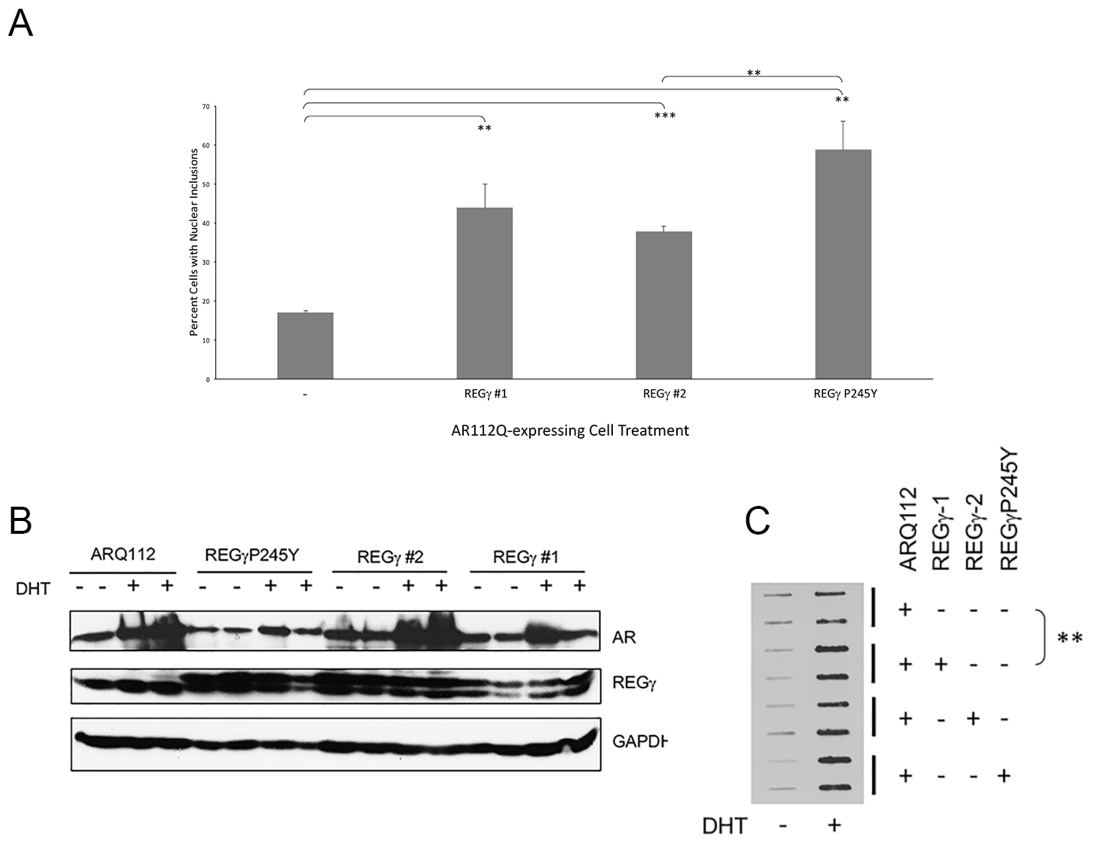

Legend to Supplemental Figure 1.

PC12 ARQ112 cells that stably overexpress either REG*γ* or REG*γ*P245Y were induced with Dox and treated with DHT for 72 hours. **A**. Cells with nuclear inclusions were visualized by immunofluorescence using AR antibody AR(H280) and quantified. **B.** Western blot analysis of REGγ and AR levels in the presence and absence of DHT. Lysates are from the same experiment shown in A. **C**, Insoluble, aggregated AR protein, isolated from the same cell populations, was detected by filter-trap assay on cellulose acetate using AR antibody AR(H280). The left column samples are without DHT (-); the right column samples are in the presence of DHT (+). Single factor ANOVA with post-hoc Bonferroni-corrected t-test was performed. ** $p < 0.01$; *** $p < 0.001$.

Supplemental Figure 2.

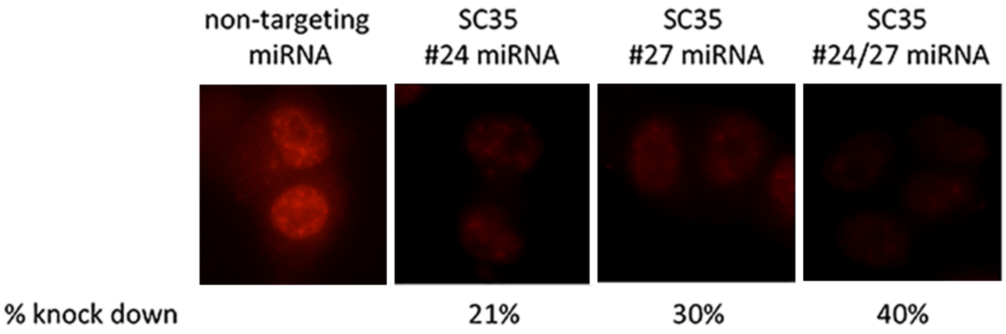

Legend to Supplemental Figure 2.

PC12 112Q cells were transfected with pcDNA™6.2-GW/ EmGFP-miR (BLOCK-iT™), containing a blasticidin resistance gene, and either SC35 #24 miR, SC35 #27 miR or co-transfected with both plasmids. After two weeks in blasticidin selection, 100% of the cells were EmGFP-positive. Cells used for the toxicity assay in Figure 9B were immunostained for SC35 and > 50 cells per condition quantified using Image J. The percent knockdown is indicated below each representative image.
